# What are the risks for Domiciliary Care Workers in Wales from COVID-19?

**DOI:** 10.23889/ijpds.v7i3.1830

**Published:** 2022-08-25

**Authors:** Rebecca Cannings-John, Fiona Lugg-Widger, Michael Robling, Simon Schoenbuchner, Hywel Jones

**Affiliations:** 1 Cardiff University

## Objectives

Domiciliary care workers (DCWs) continued to provide social care to vulnerable adults in their own homes throughout the COVID-19 pandemic. However, evidence of pandemic impact upon DCWs’ health is mixed. The OSCAR study aimed to quantify the impact of COVID-19 upon health outcomes of DCWs in Wales, and explore causes of variation.

## Approach

Data for all registered DCWs in Wales are newly available via the SAIL Databank using a secured, privacy-protecting encrypted anonymisation process. Occupational registration data for DCWs working during the pandemic was combined with electronic health records data to describe health outcomes within the first two years of the pandemic. Rates of confirmed COVID-19 infections and health outcomes including mental health contacts, fit notes, respiratory infections, and mortality will be reported and explore variation (by factors such as age, sex, ethnicity, deprivation quintile, employer). We will also explore changes over time (pre- and post- onset of COVID-19 pandemic) in outcomes.

## Results

The OSCAR study used anonymised health records for 15,727 registered DCWs in Wales. PCR-confirmed infection rates in the first full year of the pandemic (March20-February21) were 12% although lower in males (9%) than for females (12%). However, 28% of care workers received care for mental health with large differences observed between males (20%) and females (29%), and between workers from different health board regions (range 22% to 33%). The extent to which these represent pre-pandemic rates overall and how they compare to the broader community will be explored in our remaining work. A qualitative sub-study involving interviews with DCWs has informed our approach to modelling and to interpretation of findings.

## Conclusion

Using novel anonymised occupational records at a national level and existing linked EHR data and qualitative interviews, the OSCAR study will quantify the risk of COVID-19 on DCWs' health and explore sources of variation. This will provide a secure base for informing public health policy and occupational guidance.

